# Fluorescence microscopy and correlative brightfield videos of mitochondria and vesicles in H9c2 cardiomyoblasts

**DOI:** 10.1038/s41597-024-02970-5

**Published:** 2024-01-25

**Authors:** Ida S. Opstad, Åsa B. Birgisdottir, Krishna Agarwal

**Affiliations:** 1 Department of Physics and Technology, UiT The Arctic University of Norway, Tromsø, Norway; 2 Department of Clinical Medicine, UiT The Arctic University of Norway, Tromsø, Norway; 3 Division of Cardiothoracic and Respiratory Medicine, University Hospital of North Norway, Tromsø, Norway

**Keywords:** Cellular imaging, Microscopy

## Abstract

This paper presents data acquired to study the dynamics and interactions of mitochondria and subcellular vesicles in living cardiomyoblasts. The study was motivated by the importance of mitochondrial quality control and turnover in cardiovascular health. Although fluorescence microscopy is an invaluable tool, it presents several limitations. Correlative fluorescence and brightfield images (label-free) were therefore acquired with the purpose of achieving *virtual* labelling via machine learning. In comparison with the fluorescence images of mitochondria, the brightfield images show vesicles and subcellular components, providing additional insights about sub-cellular components. A large part of the data contains correlative fluorescence images of lysosomes and/or endosomes over a duration of up to 400 timepoints (>30 min). The data can be reused for biological inferences about mitochondrial and vesicular morphology, dynamics, and interactions. Furthermore, virtual labelling of mitochondria or subcellular vesicles can be achieved using these datasets. Finally, the data can inspire new imaging experiments for cellular investigations or computational developments. The data is available through two large, open datasets on DataverseNO.

## Background & Summary

Mitochondria have been the subject of numerous biomedical investigations and biological assays, due to their central importance in cell biology and energy metabolism. Their morphology and dynamics have been established as closely linked to the metabolic state of their host cell. Mitochondria can merge into large and connected networks, fragment into smaller entities or shed nano-sized particles referred to as mitochondria derived vesicles. They can remain mostly stationary as within densely packed cardiomyocytes or be transferred over large distances across and even between cells^1^.

Although each mitochondrion has its own circular DNA^2^, most of the building blocks of mitochondria are encoded in the DNA of the cell´s nucleus. Therefore, mitochondria are dependent on the cell and other cellular organelles to sustain themselves and contribute to vital processes like oxidative phosphorylation (OXPHOS). While much remains to be understood, mitochondria engage in dynamic interactions and exchange their material with e.g., endosomes and endoplasmic reticulum and undergo degradation in lysosomes^3,4^.

Analysing the morphology, dynamics and interactions between different cellular organelles is collectively of central importance towards understanding the enormous complexity of cellular biology and for deriving biomedical implications. While trying to study delicate living systems with more details and for longer duration, it is a significant challenge to assess “how the act of observing” or the assay itself might perturb the system under investigation. A certain label or amount of light exposure might be fine for one type of assay but deleterious to a different one. What is common for most biological assays relying on microscopy is the desire to reduce the possible effects of phototoxicity, cellular labels and photobleaching to a minimum.

The above-mentioned challenges together with the desire to expand our current understanding of cellular biology have motivated many computational experiments aiming to achieve *virtual labelling* (also referred to as *in silico* or *digital labelling)* of mitochondria and other cellular organelles. Virtual labelling is a computational trick to make label-free microscopy images *appear* like they have fluorescent labels with the help of machine learning engines trained on pairs of correlative label-free and fluorescence microscopy images. This approach aims at transferring the excellent specificity and contrast of fluorescence microscopy to label-free microscopy data while avoiding spurious effects of introducing exogenous components like fluorescent markers into the cells. Previously reported results in this field include Guo *et al*. employing quantitative phase microscopy with previously trained software for predicting mitochondria^5^, Ounkomol *et al*. showing label-free prediction in both DIC and brightfield 3D image stacks of DNA and nucleoli^6^, Christiansen *et al*. showing in silico labelling of various immunolabelled structures in both brightfield, phase contrast and DIC^7^ and Somani *et al*.^8^ demonstrating virtual labelling and tracking of mitochondria in brightfield 2D videos.

This data descriptor and associated datasets were created to support investigations in a similar spirit as the above-mentioned research articles. The two large datasets contain six to eight-dimensional (three spatial (XYZ), two to four channels + time) videos of H9c2 cardiomyoblasts fluorescently labelled for mitochondria, lysosomes, and endosomes in different combinations. A large portion of the data additionally contains correlative brightfield videos to support the development of label-free analysis and/or the exploration of other cellular structures than what is imaged in fluorescence mode. The datasets further contain comparable experiments for cells cultivated under both normal, glucose-rich conditions and in a glucose-deprived (galactose adapted) state. One of the open datasets described are of H9c2 cells stably transfected to express the double tag mCherry-eGFP-OMP25-TM. This double tag on the mitochondrial outer membrane is designed for monitoring mitochondrial degradation in acidic organelles like lysosomes^9^. The data is however also valuable for other studies of mitochondria, vesicles, or other cellular structures recognizable in the associated stacks of brightfield images.

In summary, we envision this data to be valuable for studies of mitochondrial dynamics, subcellular vesicles, virtual labelling of mitochondria and vesicles, and the numerous and variable interactions of mitochondria with subcellular vesicles like endosomes and lysosomes.

## Methods

### Cell culture

#### Transiently transfected cells (dataset HQDBYF)

Rat cardiomyoblast H9c2 cells (Sigma-Aldrich, 88092904) were cultured in high-glucose (4.5 g/L) Dulbecco’s Modified Eagle Medium (DMEM) with 10% Fetal Bovine Serum (FBS) and 1% streptomycin/penicillin^10^. The cells were transiently transfected for 24 hours using TransIT-LT1 (Mirus) with a plasmid encoding the trans-membrane (TM) domain of outer membrane protein 25 (OMP25), an outer mitochondrial membrane protein, fused to either mCherry or EGFP. This resulted in fluorescent labelling of the mitochondrial outer membrane.

The cells were labelled using the live-cell-friendly fluorescent marker mCLING-ATTO647N immediately before imaging using a concentration of 1:2,000 with a 12 minutes incubation time (data in folders endo_mCh-mito and endo_mCh-mito_trans) or 40 minutes with 50 nM LysoTracker Deep Red (LTDR) (data in folders endo_lyso_eGFP-mito and endo_lyso_eGFP-mito_trans). After incubation, the medium was replaced with cell-culture medium (DMEM 10% FBS) for time-lapse microscopy at 37 °C, atmospheric oxygen and 5% CO2. The membrane marker mCLING was quickly internalized by the cells and labelled small membrane-bound vesicles/endosomes inside the cells. This membrane marker exhibits a fluorescence emission maximum at a wavelength of 662 nm.

#### Stable cell-line (dataset 11LLTW)

The rat cardiomyoblast cell-line H9c2 (Sigma-Aldrich, 88092904) genetically modified using retrovirus to achieve stable expression of tandem tagged (mCherry-EGFP) mitochondrial outer membrane protein 25 (OMP 25)-transmembrane domain (TM) were utilized^11^.

Equal expression of fluorescence intensity in cells was achieved through flow cytometry-based sorting. Where indicated (galac), the cells were cultured in media without glucose but with galactose as a carbon source. The media consisted of DMEM without glucose (11966-025, Gibco) supplemented with 2 mM L-glutamine, 1 mM sodium pyruvate, 10 mM galactose, 10% FBS, 1% streptomycin/penicillin and 1 microgram/mL of puromycin (InvivoGen, ant-pr-1). The cells were adapted to galactose for a minimum of 7 days before experiments. The cells were seeded on MatTek dishes (P35G-1.5-14-C, MatTek Corporation) and imaged when they reached approximately 80% confluency.

Labelling of lysosomes (acidic endosomal system) was done by incubating the cells for 30 minutes with 50 nM Lysotracker Deep Red (LTDR, Cat nr L12492, Thermo Fisher). After labelling the media was replaced with fresh cell culture media (with either glucose or galactose in accordance with cell adaptation).

The administration of Carbonyl cyanide m-chlorophenyl hydrazone (CCCP) was done by replacing the cell culture medium on the glass bottom dish (already on the microscope) with the same medium but mixed with 10 µM CCCP. CCCP is a potent mitochondrial uncoupling agent that leads to loss of the mitochondrial membrane potential inducing changes in mitochondrial morphology. It is frequently administrated to cell culture to analyse mitochondria function as well as to induce mitochondrial stress to study removal of damaged mitochondria. More details about this experiment and the associated data files are available from the published dataset’s ReadMe file (section CCCP data in dataset 11LLTW).

### Imaging conditions

The cells were imaged at 37 °C under atmospheric gas levels.

### Imaging system

Imaging was performed using a DeltaVision OMX Blaze v4 (GE Healthcare) 3DSIM system in conventional fluorescence or transmission mode (DIC illumination mode without prism in the detection path). The system was equipped with a 60 × 1.42NA oil immersion objective and three sCMOS cameras used sequentially for fast, multi-color acquisition. An InsightSSI illumination module was used for widefield fluorescence and brightfield image acquisition. Standard excitation and emission filters for DAPI, GFP, mCherry and Cy5 were used. The centre wavelengths and other specifications of the illumination and emission filters are provided in Tables 1, 2.

**Table 1:** Illumination unit (InSightSSI) operational specifications.

Fluorophore	Color	Wavelength range (nm)	Center/ Bandpass (nm)	Nominal power (mW)	Max.
				Min.	
DAPI	Blue	381–410	395.5/29	80	140
CFP	Cyan	426–450	438/24	80	140
FITC/GFP	Green	461–493	477/32	55	110
YFP	Yellow	505–520	512.5/15	11	35
mCherry	Red	562–581	571.5/19	60	125
Cy5/DlC	Far Red	638–653	645.5/15	20	45

DIC = differential interference contrast

**Table 2:** Emission filters used in our experiments.

Channel	Center wavelength (nm)	Bandwidth (nm)
DAPI	435.5 ± 2.0	31.0 ± 2.0
FITC	528.0 ± 2.0	48.0 ± 2.0
mCherry, Alexa Fluor 568	609.0 ± 2.0	37.0 ± 2.0
Cy5	683.0 ± 2.0	40.0 ± 2.0
CFP	477.5 ± 2.0	35.0 ± 2.0
YFP	541.0 ± 2.0	22.0 ± 2.0

### Image acquisition

Multi-color z-stacks were acquired using a z-spacing of either 250 nm or 500 nm and 80 nm XY pixel size. Image intensities and exposure times were kept low but adjusted high enough to ensure satisfactory image quality. The individual acquisition settings for all images are available in the associated log files on DataverseNO. Additionally, an overview of the dimensionality of all available files are provided in Tabel 3 and Tabel 4 (Excel sheets are available in the published datasets).

### Data processing

#### Image registration

Up to three different cameras were used for fast multi-channel acquisition. Therefore, image registration (channel alignment) was performed using the proprietary software supplied with the microscope DeltaVision OMX Blaze v4 (ALX in the image files indicate channel alignment). This ensures that the XYZ location for different channels in the same data file accurately corresponds to the same sample location.

#### Deconvolution

The files with names appended _D3D have been deconvolved in three dimensions using the proprietary SoftWoRx software supplied with the microscope. The processing was constrained iterative deconvolution with additive gaussian blur and using an experimentally obtained point spread function of the imaging system. Software deconvolution options applied: *Method*: Enhanced Ratio (aggressive); *Number of Cycles*: 10; *Noise Filtering*: Medium (200 nm).

#### Z-projection

The files with names appended _PRJ have been z-projected for maximum intensity using either the microscope’s proprietary software or ImageJ/Fiji^12^. These files are useful directly in some image analysis pipelines or just to obtain an overview of the data without downloading or opening the full three-dimensional image data.

#### Compressed videos

The AVI files are small videos compared to both the raw and PRJ data and can be played to quickly visualize multi-color subcellular dynamics without downloading large files or using any specialized software. They are generated in Fiji using maximum intensity projection and combined view of the fluorescence channels. The AVI files are saved using JPEG compression and 3 fps.

## Data Records

**Fig. 1:**
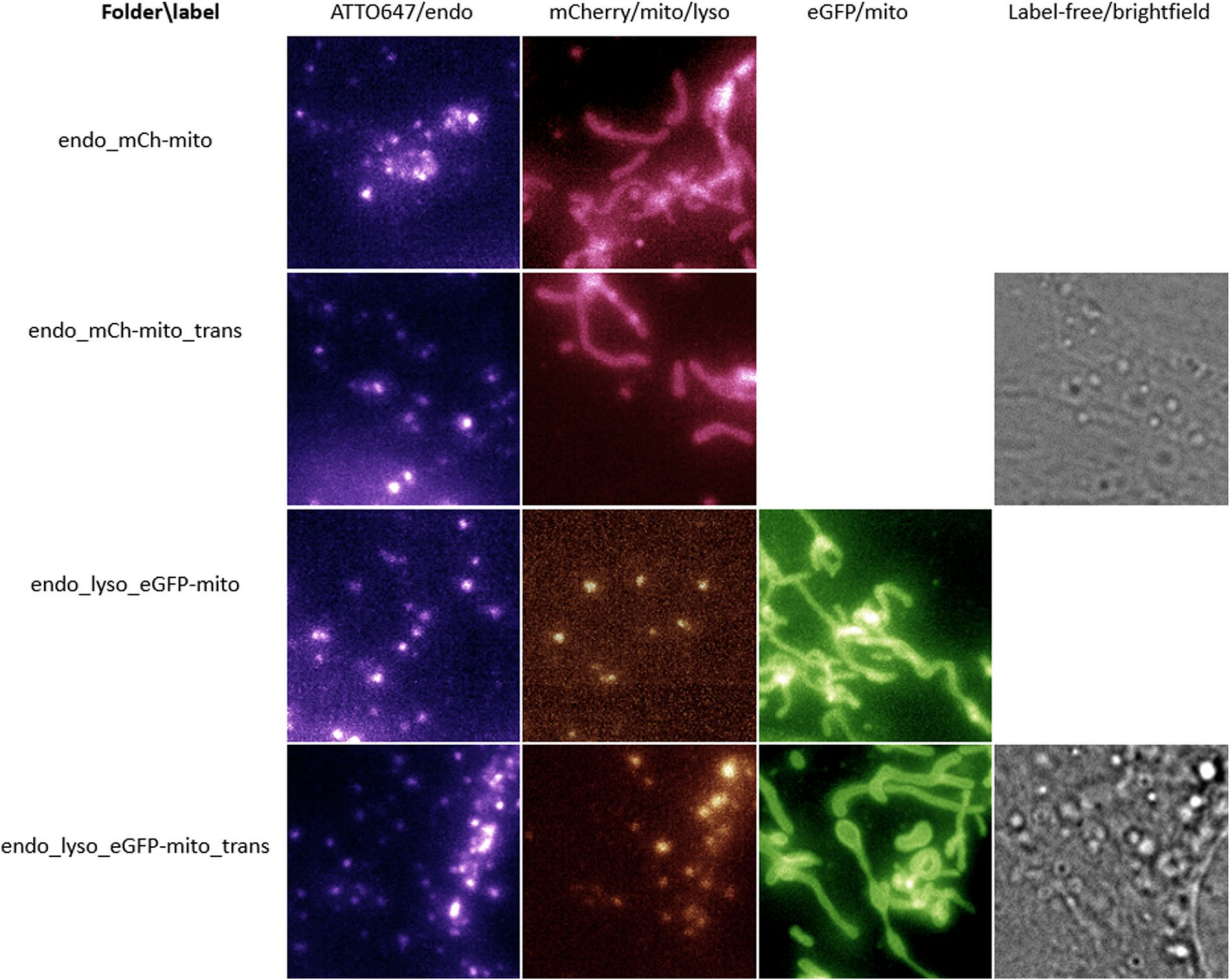
Overview of dataset HQDBYF of transiently transfected H9c2 cardiomyoblasts labelled for mitochondria, endosomes and (where indicated) lysosomes. Two of the folders additionally provide correlative brightfield images as a separate channel.

**Fig. 2:**
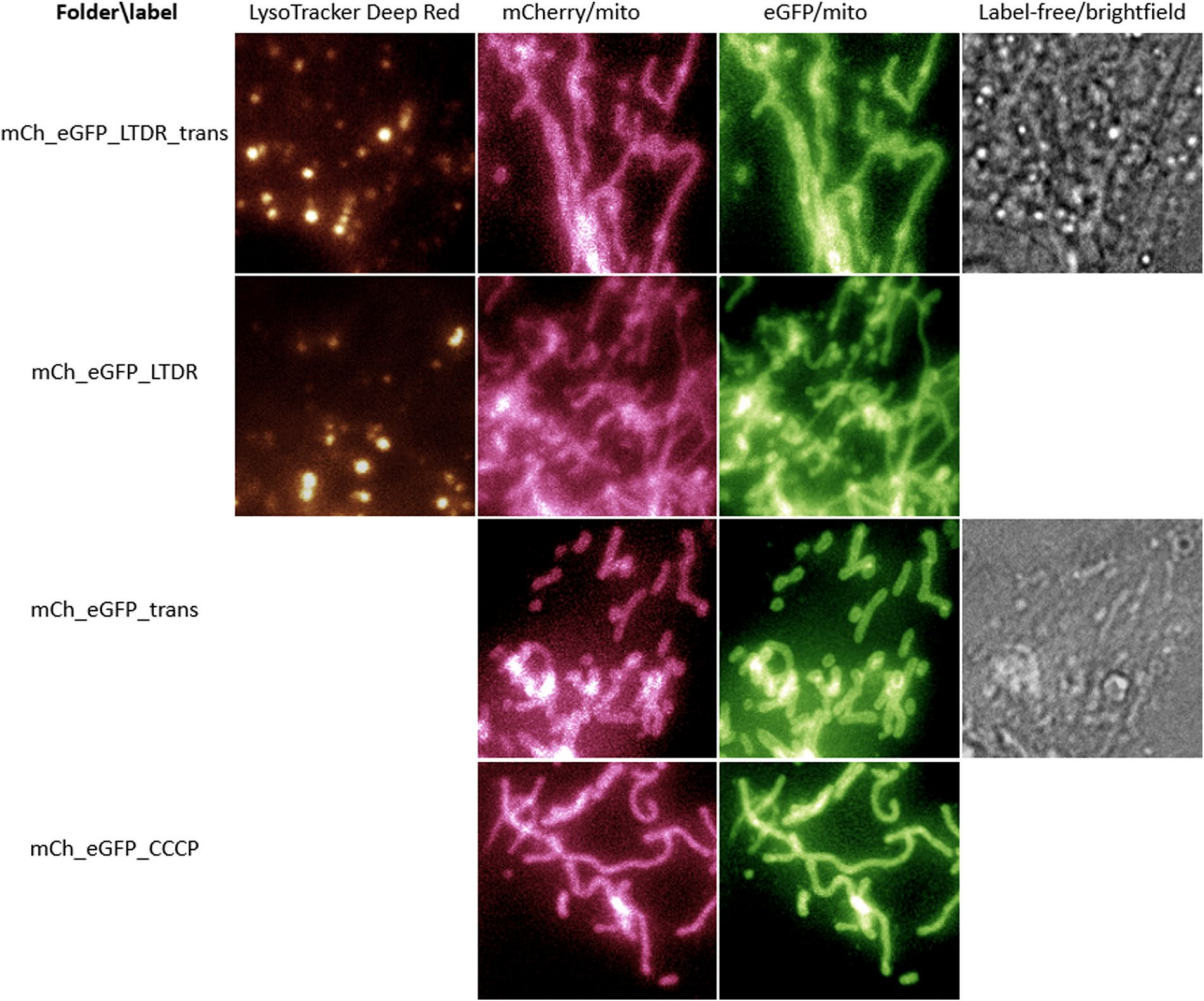
Overview of dataset 11LLTW of H9c2 cardiomyoblasts with a stable double tag for the mitochondrial outer membrane. Where indicated the cells are further labelled for lysosomes and/or have correlative brightfield images. The double tag can be used to study acidic mitochondria and interactions with acidic organelles.

The data is available from two open datasets on the DataverseNO repository in the UiT Open Research Data collection: *Fluorescence microscopy videos of mitochondria and endosomes in H9c2 cardiomyoblasts* (transient cells, identifier: HQDBYF)^10^ and *Fluorescence microscopy videos of mitochondria in H9c2 cardiomyoblasts* (stable cells with double tag, identifier: 11LLTW)^11^. The data is divided into subfolders according to experimental conditions and imaging mode as visualized in Fig. 1 (data HQDBYF) and Fig. 2 (data 11LLTW).

### File formats

The image files are either TIFF or DV (DeltaVision’s format) and can be opened using e.g. the open source freeware ImageJ/Fiji^12^. The log files can be open in any text editor.

### Folder naming

Folders with endo_ contain fluorescence images of endosomes (visualized using the membrane marker mCLING-ATTO647n). Folders with _mCh-mito contain images of mitochondria with transient mCherry (a red fluorescent protein) expression. Folders with _ eGFP-mito contain images of mitochondria with transient eGFP expression (enhanced green fluorescent protein). The folders with _lyso contain fluorescent images of lysosomes (labelled using LysoTracker Red). Folders with _trans contain correlative (transmission) brightfield images (available as the last channel in the multi-color images). Except for being exposed additionally to light for the brightfield image, this data has the same experimental conditions as the corresponding folder without _trans but are of different cells avoiding duplication of data. Folders with mCh_eGFP contain images of both red and green fluorescence of dually labelled mitochondria (the double tag to recognize acidic mitochondria). Folders with _LTDR contain images of lysosomes labelled using LysoTracker Deep Red. The folder with _CCCP contains data from the experiment using CCCP.

More details about the experimental conditions, subfolders, overview of the file names together with an explanation of the file naming convention are available in the ReadMe files of the published datasets.

## Technical Validation

### Imaging system and data acquisition

The commercial OMX Blaze 3DSIM imaging system (for this data only used in conventional imaging mode) was installed and quality checked by a system engineer. The immersion oil was chosen to provide the imaging system with a symmetric three-dimensional point spread function. The quality of image registration was ensured by using an image registration file of similar data as the relevant experiments. This registration file was generated using a sample from the microscope’s manufacturer together with the microscope’s proprietary software for image registration. The data and channel alignment were further manually checked using the tandem-tagged samples where mitochondria were visible in all three channels. Here, three different cameras were used with emission filters for RFP, GFP and DAPI, where the DAPI channel/camera was used for brightfield.

### Cell-line

For retroviral-based creation of stable cells with constitutive expression of fluorescent mitochondria marker, we used the commercially available and authenticated cell-line H9c2 (2-1) Rat DB1X heart myoblast (Catalogue No: 88092904; Lot No. 08G008 P + 8; European Collection of Authenticated Cell Cultures; Distributed by Sigma Aldrich, Norway). To ensure uniform fluorescent marker expression, the cells were sorted using flow cytometry. The cells tested negative for mycoplasma.

### Biological context

Glucose serves as the primary metabolic fuel of mammals^13^. Glucose is converted to energy in the form of adenosine triphosphate (ATP) in a process termed glycolysis. The main ATP energy production in normal cells, however, is conducted in the mitochondria through oxidative phosphorylation (OXPHOS). H9c2 cells under normal culture conditions with high glucose levels primarily utilize glycolysis for ATP generation. In contrast, H9c2 cells deprived of glucose and adapted to galactose rely entirely on OXPHOS to meet their energy requirements. Consequently, glucose deprivation has been shown to render the cells more sensitive to mitochondrial damage^14^. This is likely to cause differences between cells under the two different cultivation conditions, although we have also noted a large variation within the two groups when it comes to e.g., mitochondrial morphology, number of lysosomes, and number of mitochondrial fragments found in lysosomes. In reference^15^, the glucose-deprived cells were shown to contain more mitochondria-derived vesicles than the cells given glucose (i.e., normal growth conditions).

## Usage Notes

### Files and data

The images (TIFF and DV files) can be opened in ImageJ/Fiji or other programs for image processing. The acquisition logs or other text files (log or txt) can be opened using any text editor. Many of the larger image files are zip and must be decompressed before use (e.g. using 7-zip or similar software). An overview of the image content and biological events can be quickly accessed through the AVI or _PRJ files. The experimental meaning of folder and file names are explained in the datasets’ ReadMe files (in the DATA & FILE OVERVIEW sections). The experimental settings for each image file can be accessed without opening the images via the text files with the same name.

The datasets contain large image data of up to 400 multichannel volumetric timepoints, resulting in a size of up to tens of GBs per file. A fast and stable internet connection is thus recommended. We also advice using the AVI or z-projected files (_PRJ) for prior assessment during the search for suitable image data.

**Fig. 3:**
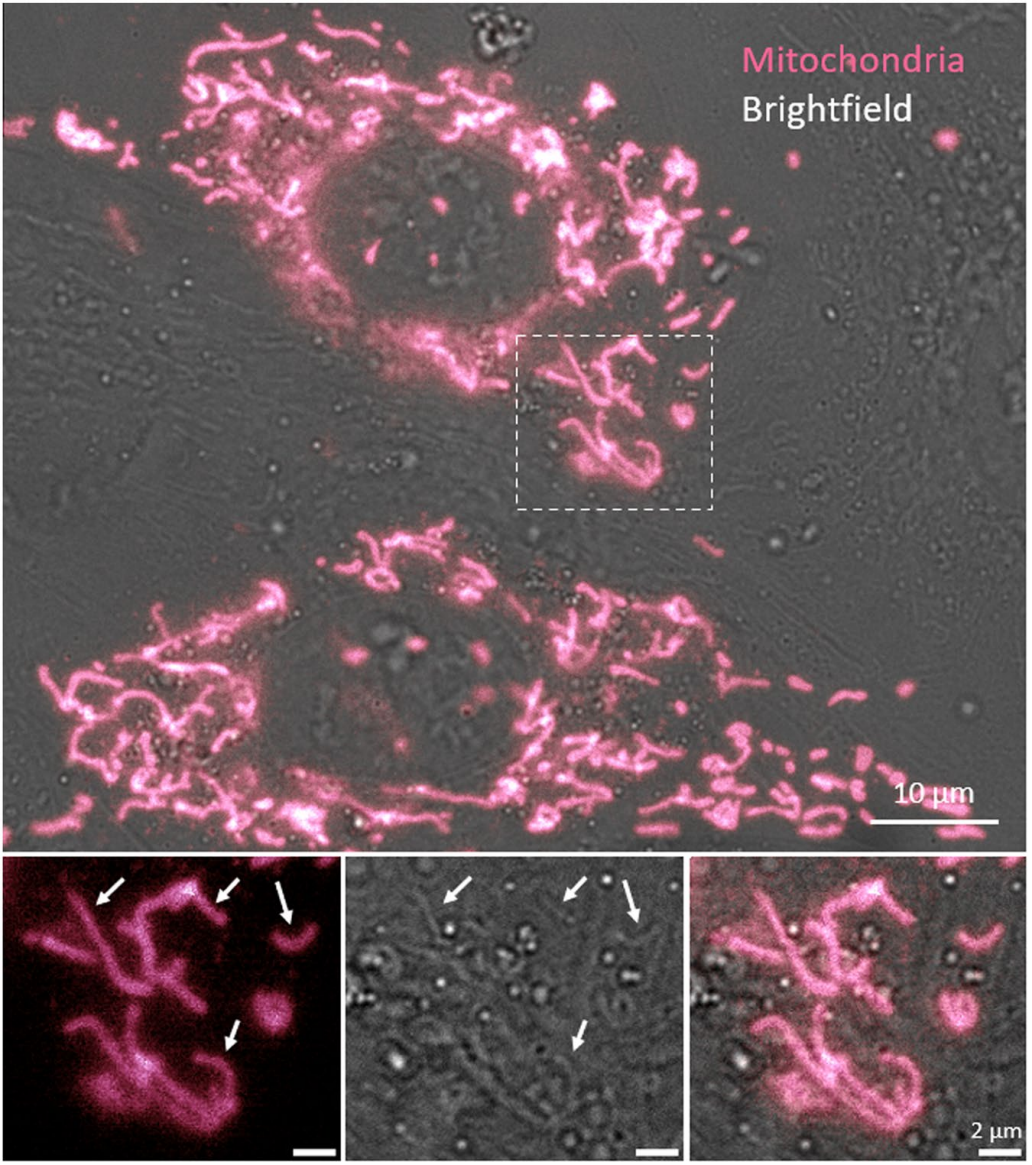
Combined brightfield and fluorescence channel of mCherry-tagged mitochondria in transiently transfected H9c2 cardiomyoblasts. The arrows indicate mitochondria that can easily be recognised in both the fluorescence and brightfield views. Also note the presence of unlabelled cells/mitochondria towards the left and right sides. This can have negative implications if applying this data in the training procedure for digital/virtual labelling of mitochondria. To train a classifier for virtual labelling of mitochondria, it is better to use a stable cell-line (as in the dataset 11LLTW), where all cells display a uniform expression of the mitochondrial marker. For applying a trained classifier, training of classifiers for labelled vesicles or studies of organelle morphology and dynamics, the data of transiently labelled cells is still recommended.

If the data is to be used for the training of a classifier for virtual labelling of mitochondria, it is best to use dataset 11LLTW of stably labelled mitochondria. In the transiently labelled samples, not all cells are expressing the fluorescent mitochondrial marker (illustrated in Fig. 3), and this can confuse the training algorithm if not proper care is taken. A way to account for the uncomplete fluorescence labelling of mitochondria can be to split the large (1024 × 1024 pixels) raw images into smaller images and then selecting only the sub-images with above a certain threshold of fluorescence for classifier training. For example, in Somani *et al*.^8^ only sub-images with a proportion of detected mitochondria >20% were used for classifier training.

The transient cell data (HQDBYF) is particularly suitable for training of vesicle classification, application of already trained virtual labelling software or other general analysis of mitochondrial and vesicle dynamics, interactions, and morphology.

Both the fluorescence and correlative brightfield data are provided as three-dimensional stacks (three spatial dimensions in addition to time and channels). Single planes can be extracted for analysis. For virtual labelling, however, and for many other applications, using the entire image stacks is likely to give a better performance as especially in the brightfield data, cellular structures from all image planes blend together and it is a very hard classification problem to separate the noisy structures correctly in the axial dimension^8^.

Many machine learning algorithms require a huge amount of varied data to be suitably trained for a certain or a diverse set of image classification tasks. Individual image planes and time-points can in some case be used as individual images, but we caution the readers to be aware of the danger of overfitting, as the similarities between images of different time-points of the same cell can be very high. The mitochondria and vesicles image are however very dynamic organelles and almost continuously change their shape and/or location. Different timepoints of the same sample region might therefore successfully be used as individual sample in various training algorithms if the time between each image is sufficient. If for example every tenth image is used from a time-lapse, the images will be much less correlated than using every frame of the time-lapse. The time between frames is shown in Table 3 and Table 4 (columns names *dT*) as well as the total number of frames available for each data file.

**Table 3:** Overview of image files and dimensionality of the dataset HQDBYF.

Filename	Dimensions	Brightfield	dT (s)	Z span (µm)	Z planes	dZ (µm)
**HQDBYF: transient cell lines**						
endo_lyso_eGFP_mito(_trans)\						
20190717_H9c2-G3_eGFP-OMP_LTR50nM_mC1to2000-40min_1520_convTL_002	3D-3C-50T	no	2	5.5	23	0.25
20190717_H9c2-G3_eGFP-OMP_LTR50nM_mC1to2000-40min_1520_convtTL_004	3D-4C-100T	yes	2	4	17	0.25
20190717_H9c2-G3_eGFP-OMP_LTR50nM_mC1to2000-40min_1520_convTL_005	3D-3C-100T	no	2	4	17	0.25
20190717_H9c2-G3_eGFP-OMP_LTR50nM_mC1to2000-40min_1520_convtTL_006	3D-4C-25T	yes	5	4	17	0.25
20190717_H9c2-G3_eGFP-OMP_LTR50nM_mC1to2000-40min_1520_convtTL_008	3D-4C-25T	yes	5	7	29	0.25
endo_mCh_mito(_trans)\						
20190717_H9c2-G2_mCh-OMP_inFBS_mC1to2000-10min_1520_convTL_002	3D-2C-200T	no	2	4	17	0.25
20190717_H9c2-G2_mCh-OMP_inFBS_mC1to2000-10min_1520_convTL_003	3D-2C-400T	no	2	4	17	0.25
20190717_H9c2-G2_mCh-OMP_inFBS_mC1to2000-10min_1520_convtTL_004	3D-3C-400T	yes	2	4	17	0.25
20190717_H9c2-G2_mCh-OMP_inFBS_mC1to2000-10min_1520_convtTL_005	3D-3C-400T	yes	2	4	17	0.25
20190717_H9c2-G2_mCh-OMP_inFBS_mC1to2000-10min_1520_convtTL_006	3D-2C-200T	yes	2	3	13	0.25
20190717_H9c2-G2_mCh-OMP_inFBS_mC1to2000-10min_1520_convtTL_007	3D-3C-300T	yes	2	3.5	15	0.25
20190717_H9c2-G2_mCh-OMP_inFBS_mC1to2000-10min_1520_convtTL_008	3D-3C-300T	yes	5	3	13	0.25

For example, 3D-3C-50T, refers to three spatial dimensions (XYZ), three color channels and 50 time-points. The column Brightfield indicates whether correlative label-free images are available, dT is the time between each frame, Z span the image volume’s axial span or thickness, Z planes the number of sections in the volume and dZ the spacing between each section in micrometres.

**Table 4:** Overview of image files and dimensionality of the dataset 11LLTW.

Filename	Dimensions	Brightfield	dT (s)	Z span (µm)	Z planes	dZ (µm)
**11LLTW: stable cell line**						
mCh_eGFP_CCCP\cell1\						
20210611_H9c2-dTag_CCCP10uM_1518_conT0_001_ALX.dv	3D-2C-1T	no	30	3	7	0.5
20210611_H9c2-dTag_CCCP10uM_1518_conTL_002_ALX	3D-2C-53T	no	30	4	9	0.5
20210611_H9c2-dTag_CCCP10uM_1518_conTL_002b_ALX	3D-2C-61T	no	30	4	9	0.5
mCh_eGFP_CCCP\cell2\						
20210611_H9c2-dTag_normal_1518_conTL_001_ALX	3D-2C-61T	no	30	4	9	0.5
20210611_H9c2-dTag_CCCP10uM_1518_conTL_002_ALX	3D-2C-61T	no	30	4	9	0.5
20210611_H9c2-dTag_CCCP10uM_1518_conTL_003_ALX	3D-2C-61T	no	30	4	9	0.5
mCh_eGFP_LTDR\						
20200227_H9c2-galac-mCh-eGFP_LTDR50nM-30min_1522_dvtl_003_ALX_PRJ.dv	3D-3C-50T	no	5	2	5	0.5
20200227_H9c2-galac-mCh-eGFP_LTDR50nM-30min_1522_dvtl_007_ALX_PRJ.dv	3D-3C-100T	no	5	2	5	0.5
20200227_H9c2-galac-mCh-eGFP_LTDR50nM-30min_1522_dvtl_008_ALX_PRJ.dv	3D-3C-61T	no	5	2	5	0.5
20200227_H9c2-galac-mCh-eGFP_LTDR50nM-30min_1522_dvtl_011_ALX_PRJ.dv	3D-3C-61T	no	5	2	5	0.5
20200227_H9c2-galac-mCh-eGFP_LTDR50nM-30min_1522_dvtl_0014_ALX_PRJ.dv	3D-3C-61T	no	5	2	5	0.5
20200227_H9c2-galac-mCh-eGFP_LTDR50nM-30min_1522_dvtl_0014_ALX_PRJ.dv	3D-3C-100T	no	5	2	5	0.5
mCh_eGFP_LTDR_trans\galac\						
20210330_H9c2_mito-mChGFP_LTRDR50nM_1522_dvt-5sTL_002_ALX_PRJ	3D-4C-40T	yes	5	2	8	0.25
20210330_H9c2_mito-mChGFP_LTRDR50nM_1522_dvt-5sTL_003_ALX_PRJ	3D-4C-60T	yes	5	2	8	0.25
20210330_H9c2_mito-mChGFP_LTRDR50nM_1522_dvt-5sTL_004_ALX_PRJ	3D-4C-60T	yes	5	2	8	0.25
20210330_H9c2_mito-mChGFP_LTRDR50nM_1522_dvt-5sTL_005_ALX_PRJ	3D-4C-60T	yes	5	2	8	0.25
20210330_H9c2_mito-mChGFP_LTRDR50nM_1522_dvt-5sTL_006_ALX_PRJ	3D-4C-60T	yes	5	2	8	0.25
20210330_H9c2_mito-mChGFP_LTRDR50nM_1522_dvt-5sTL_007_ALX_PRJ	3D-4C-60T	yes	5	2	8	0.25
20210330_H9c2_mito-mChGFP_LTRDR50nM_1522_dvt-5sTL_008_ALX_PRJ	3D-4C-60T	yes	5	2	8	0.25
mCh_eGFP_LTDR_trans\gluc\						
20210330_H9c2-gluc_mito-mChGFP_LTRDR50nM_1522_dvt-5sTL_003	3D-4C-60T	yes	5	2	8	0.25
20210330_H9c2-gluc_mito-mChGFP_LTRDR50nM_1522_dvt-5sTL_005	3D-4C-60T	yes	5	2	8	0.25
20210330_H9c2-gluc_mito-mChGFP_LTRDR50nM_1522_dvt-5sTL_006	3D-4C-60T	yes	5	2	8	0.25
20210330_H9c2-gluc_mito-mChGFP_LTRDR50nM_1522_dvt-5sTL_007	3D-4C-60T	yes	5	2	8	0.25
20210330_H9c2-gluc_mito-mChGFP_LTRDR50nM_1522_dvt-5sTL_008	3D-4C-60T	yes	5	2	8	0.25
20210330_H9c2-gluc_mito-mChGFP_LTRDR50nM_1522_dvt-5sTL_009	3D-4C-60T	yes	5	2	8	0.25
mCh_eGFP_trans\						
20201008_H9C2_mCherry-EGFP-OMP25_galac_37dg-CO2_1520_TL_008	3D-3C-200T	yes	3	8	33	0.25
20201008_H9C2_mCherry-EGFP-OMP25_galac_37dg-CO2_1520_TL_009	3D-3C-200T	yes	3	8	33	0.25
20201008_H9C2_mCherry-EGFP-OMP25_galac_37dg-CO2_1520_TL_010	3D-3C-200T	yes	3	8	33	0.25
20201008_H9C2_mCherry-EGFP-OMP25_galac_37dg-CO2_1520_TL_011	3D-3C-200T	yes	3	8	33	0.25
20201008_H9C2_mCherry-EGFP-OMP25_galac_37dg-CO2_1520_TL_012	3D-2C-500T	yes	3	8	33	0.25

Each raw image file is of approximately one large cardiomyoblast, but each cell typically contains hundreds of mitochondria and vesicles. Each image can therefore be split into several sub-images for some types of image analysis or classifier training. For example, in Somani *et al*.^8^, each 1024 × 1024 pixels image plane was split into 25 sub-images for classifier training. Additionally, each z-section of the 3D volumes was treated as individual images. Following a similar procedure as in^8^, the 3D time-lapses provided should suffice for several hundred thousands of samples for classifier training for virtual labelling.

**Fig. 4:**
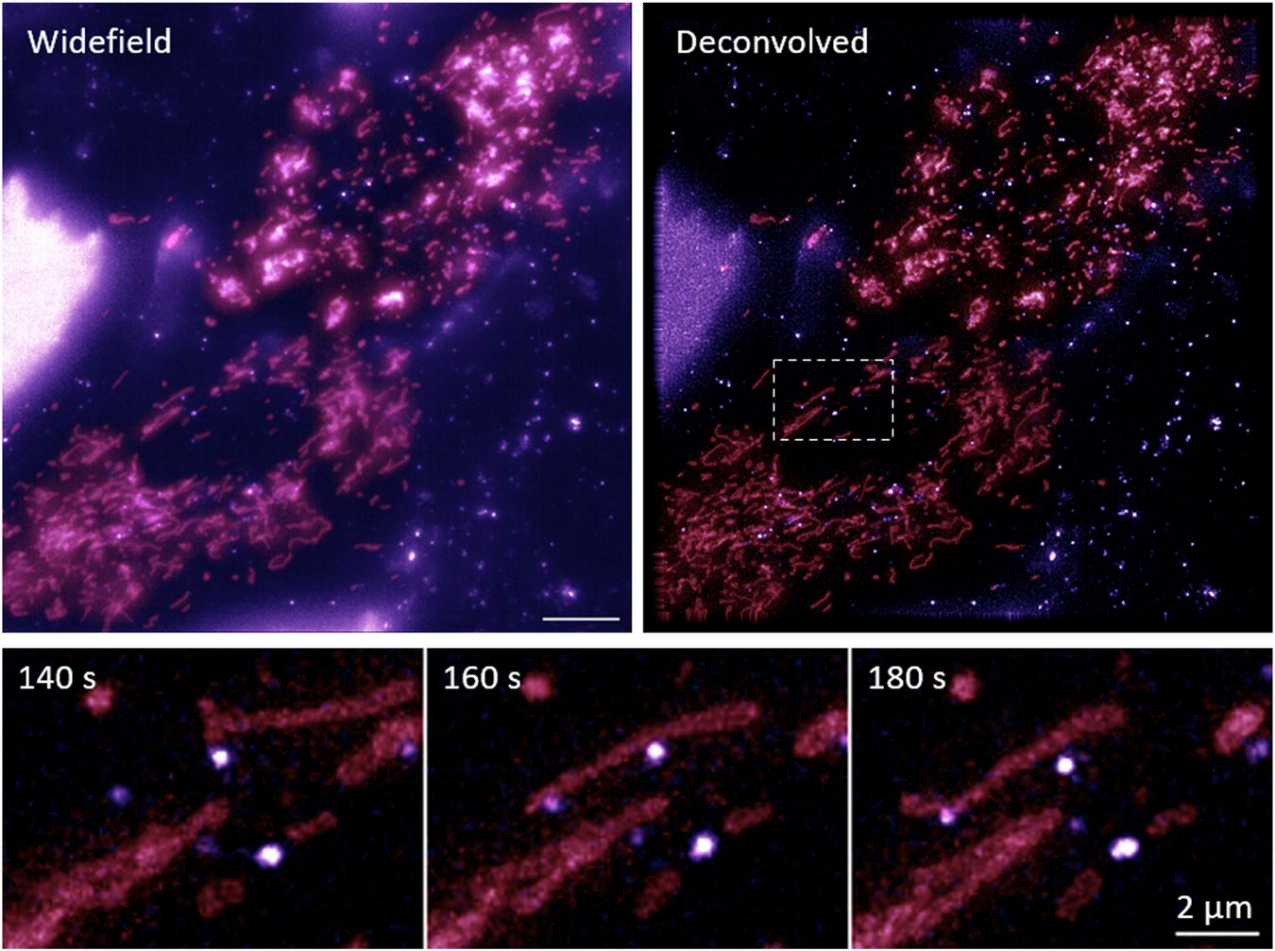
Deconvolved image data is provided to ease the visual analysis of endosome dynamics and interactions with mitochondria. The images displayed are maximum intensity z-projections of the volumetric image data. The data is available from the DataverseNO dataset HQDBYF^10^.

For the dataset with tagged endosomes (HQDBYF) also deconvolved images (in addition to the unprocessed widefield images) are provided to aid especially the visual analysis of endosome-mitochondria interactions and dynamics. A comparison of a normal fluorescence and corresponding deconvolved image is provided in Fig. 4. The lower row depicts endosome- mitochondria interactions and dynamics at improved contrast after deconvolution. Although slightly blurred, the unprocessed data is still preferred for types of analysis taking the systematic blurring of the microscope into account, as e.g. described for the physics-based segmentation described in Sekh *et al*.^16^.

**Fig. 5:**
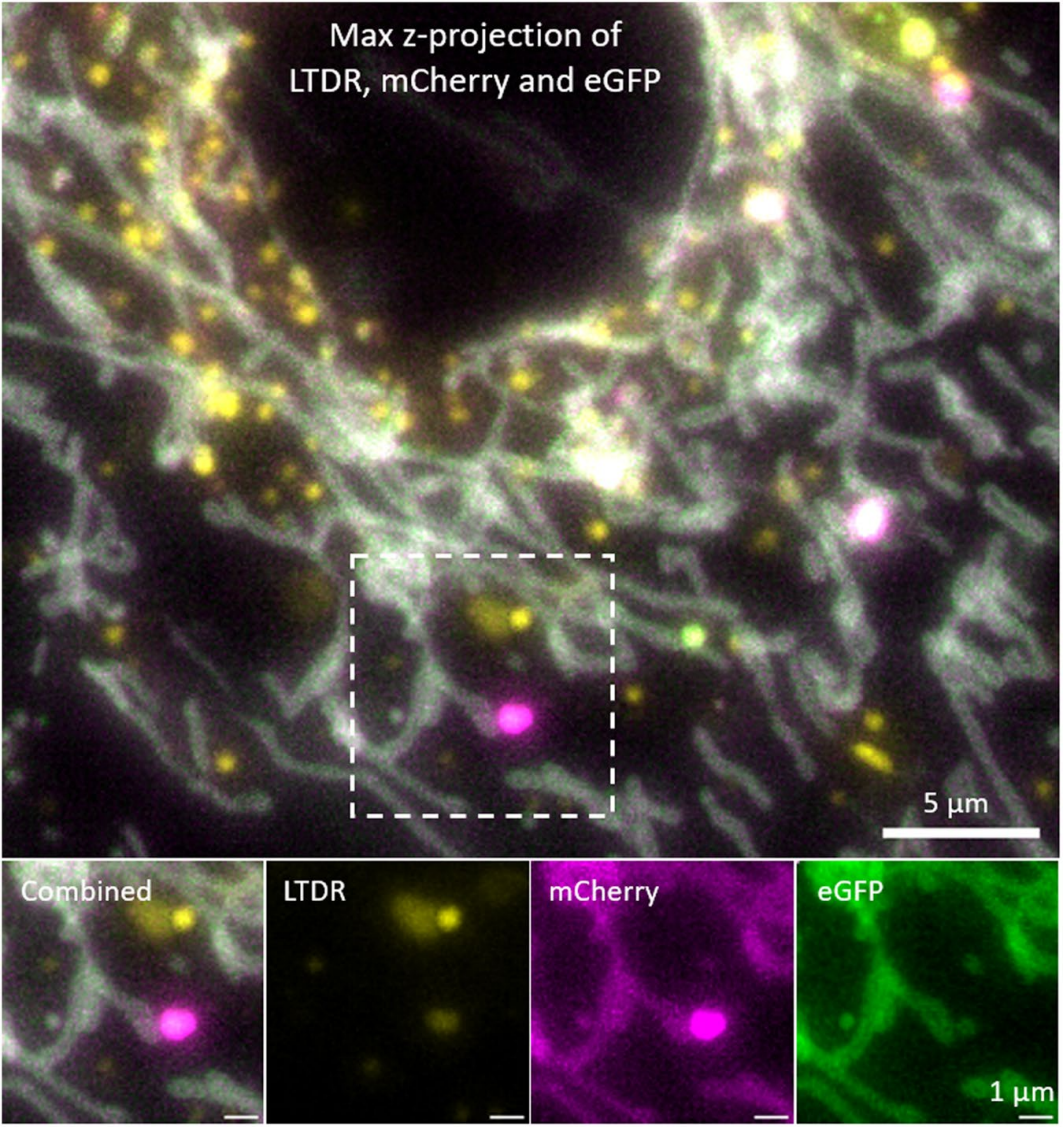
Detecting mitochondrial degradation in acidic vesicles using the tandem tag mCherry-eGFP and LysoTracker Deep Red. Red (in figure magenta) spots in the combined image indicate the accumulation and degradation of mitochondria in lysosomes. LTDR signal indicated the presence of an acidic compartment and the relative (to red) low green signal indicated degradation of mitochondria. This is because of the high stability of mCherry in low pH compared to eGFP.

The dataset 11LLTW are of H9c2 cells expressing the acidity sensor mCherry-eGFP on the outer mitochondrial membrane. This cell-line is designed for monitoring mitochondrial degradation in acidic organelles like lysosomes. How this works in practice is illustrated in Fig. 5. The high stability of mCherry compared to eGFP in low pH environments, render “red-only” spots in the combined fluorescence images as indicators of mitochondrial degradation in lysosomes. More details on its use and quantification is available from a recent work by Gustav Godtliebsen *et al*.^17^.

As a final note, we envision that this dataset can be used for valuable biological inferences on its own, but also in combination with other published datasets and to guide and inspire new imaging experiments for cellular investigations.

## Data Availability

The image deconvolutions and associated image registrations were completed using the proprietary software SoftWoRx 7.0.0 following the microscope.
